# As if sand were stone. New concepts and metrics to probe the ground on which to build trustable AI

**DOI:** 10.1186/s12911-020-01224-9

**Published:** 2020-09-11

**Authors:** Federico Cabitza, Andrea Campagner, Luca Maria Sconfienza

**Affiliations:** 1 Dipartimento di Informatica, Sistemistica e Comunicazione, Universitá degli Studi di Milano-Bicocca, Viale Sarca, 336, Milan, 20125 Italy; 2 IRCCS Istituto Ortopedico Galeazzi, Via Riccardo Galeazzi 4, Milan, 20161 Italy; 3 Department of Biomedical Sciences for Health, Università degli Studi di Milano, Via Mangiagalli 31, Milan, 20133 Italy

**Keywords:** Gold standard, Explainable AI, Machine learning, Reliability, Usable AI

## Abstract

**Background:**

We focus on the importance of interpreting the quality of the labeling used as the input of predictive models to understand the reliability of their output in support of human decision-making, especially in critical domains, such as medicine.

**Methods:**

Accordingly, we propose a framework distinguishing the reference labeling (or Gold Standard) from the set of annotations from which it is usually derived (the Diamond Standard). We define a set of quality dimensions and related metrics: representativeness (are the available data representative of its reference population?); reliability (do the raters agree with each other in their ratings?); and accuracy (are the raters’ annotations a true representation?). The metrics for these dimensions are, respectively, the *degree of correspondence*, *Ψ*, the *degree of weighted concordance* *ϱ*, and the *degree of fineness*, *Φ*. We apply and evaluate these metrics in a diagnostic user study involving 13 radiologists.

**Results:**

We evaluate *Ψ* against hypothesis-testing techniques, highlighting that our metrics can better evaluate distribution similarity in high-dimensional spaces. We discuss how *Ψ* could be used to assess the reliability of new predictions or for train-test selection. We report the value of *ϱ* for our case study and compare it with traditional reliability metrics, highlighting both their theoretical properties and the reasons that they differ. Then, we report the *degree of fineness* as an estimate of the accuracy of the collected annotations and discuss the relationship between this latter degree and the *degree of weighted concordance*, which we find to be moderately but significantly correlated. Finally, we discuss the implications of the proposed dimensions and metrics with respect to the context of Explainable Artificial Intelligence (XAI).

**Conclusion:**

We propose different dimensions and related metrics to assess the quality of the datasets used to build predictive models and Medical Artificial Intelligence (MAI). We argue that the proposed metrics are feasible for application in real-world settings for the continuous development of trustable and interpretable MAI systems.

## Background

This study contributes to the assessment of the trustworthiness of medical decision support systems built using Machine Learning (ML) techniques, an instance of so-called Medical Artificial Intelligence (MAI) [1]. Thus, we begin with a general question: “when can we call a decision support trustable?”. Trust in technology is a vast research topic (e.g., [2]), but we can ground our approach on an intuitive notion of it: we trust an advisor (and hence are willing to rely on his advice) if his *reputation* is good; if we generally agree with his recommendations (i.e., we find them plausible); if he convinces us that he is right (or persuasiveness); and if we think his sources and knowledge are good (or expertise). These intuitive notions have clear counterparts in the MAI domain: reputation relates to *accuracy* (on past cases); plausibility to *human-machine concordance*; persuasiveness relates to *explainability*, or better yet, to *causability* [3]; and the advisor’s expertise relates to what one of the founders of ML evocatively called the *experience* of the ML system [4] (p.2).

In this paper, we focus on this last^1^ characteristic of a trustable agent, the quality of the “experience,” on the basis of which this agent learns how to recognize and classify new instances; that is, the quality of the available training dataset, which contains the so-called *Gold Standard* (i.e., the set of target labels associated with the data).

We interpret the quality of the MAI support in terms of three complementary aspects, and we propose a novel metric for each aspect (see Table 1):

1. the extent the MAI’s training set is representative, with respect to a reference population (or a random sample drawn from it), in terms of its *degree of correspondence*. This degree can also be used to assess the extent to which the MAI’s “experience” (in the sense above) is compatible with any new case in which it is supposed to give its advice.

**Table 1:** A summary of the dimensions proposed, the related metrics to measure them, and the information required to compute the metrics

Dimension	Metrics	Required Information
Representativeness	Degree of correspondance, *Ψ*	Two datasets of arbitrary size
Reliability	degree of weighted concordance *ϱ*	Raters’ accuracy and confidence on each rating
Gold Standard Accuracy	*degree of fineness*, *ϕ*	Accuracy of the raters, type of reduction
2. the extent the Gold Standard is accurate with respect to the theoretically correct labels (which, in most cases, is unknown) in terms of an estimate, which we call *degree of fineness*;
3. and, in the advisable case where the Gold Standard is derived from a set of annotations by multiple raters (what we call *Diamond Standard*), the extent to which the Gold Standard is reliable, evaluated in terms of the *degree of weighted concordance* [5] of its Diamond Standard. We will also explain why the multi-rater case is more advisable than the single-rater one and why the use of more raters is better.

Although the topic of the reliability (in the broadest sense) of the Gold Standard is seemingly consolidated, we will make the point that it is in fact overly neglected, and its impact on the overall quality of an MAI is greatly underrated.

We pose the following research questions: How *accurate* and *reliable* is the Gold Standard? How *informative* (or *representative*) are the training data with respect to the reference population and to any new case where we want support from technology? And, related to this aspect, how similar are two datasets? That is, how we can be confident that the good performance of a model that has been validated on one dataset will be reproduced when fed with cases from the other dataset?

**Fig. 1:**
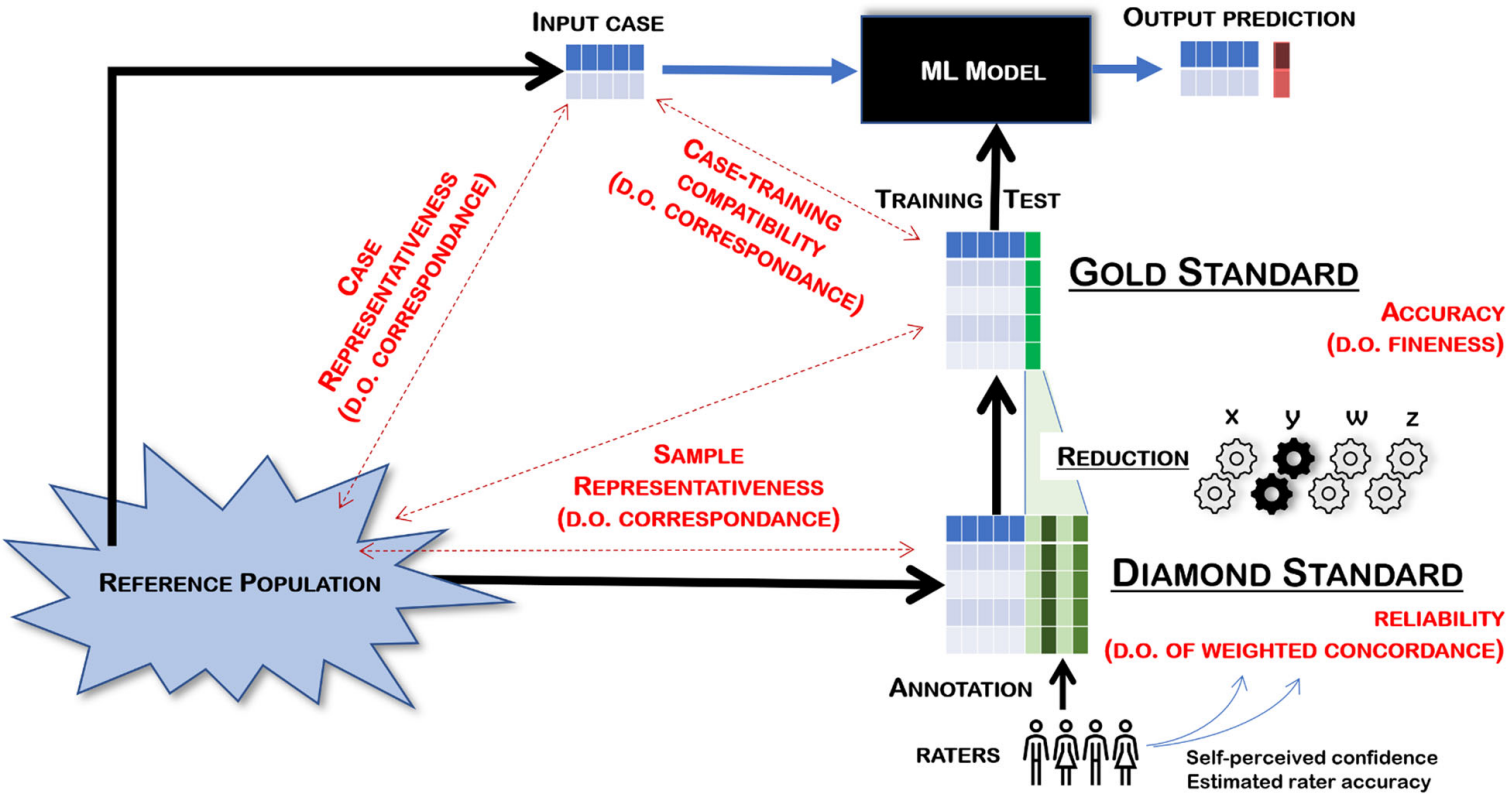
The general framework and the main concepts illustrated in this contribution. The underlined words represent the proposed data structures and procedures; the red words represent the quality dimensions and metrics to measure them. This image was generated using Python and the Matplotlib library (v. 3.3.0), like all other images in this article

To address these questions, we propose a general framework to circumscribe the main concepts related to the quality of the data feeding the Machine Learning process. With reference to Fig. 1, the *Gold Standard* is the available “experience” upon which the ML model is trained, that is, the set of labels associated with the cases represented in the training set. Thus, each case is labeled with a unique value for the target feature. We therefore distinguish the Gold Standard from what we call the *Diamond Standard*: the collection of annotations that *m* annotators (also called raters or observers) have associated with the cases in the training set.

We then use the term *reduction* to refer to the data transformation that produces the Gold Standard from the Diamond Standard. Reductions necessarily entail some information loss because they allow a shift from multi-rater labeling to “the one best” type of labeling by a “collective” rater (usually, but not necessarily always representing the majority of the raters involved). Obviously, if *m* (the number of raters) is equal to 1, the Gold Standard and the Diamond Standard coincide.

We will discuss how to estimate the quality of the Gold Standard on the basis of the type of reduction used to generate it from the Diamond Standard and the reliability of this latter. This reliability, in turn, is assessed based on the number and interpretative skills of the annotators involved, that is, their accuracy and their confidence in their choices. In other words, if the *ground truthing* process is usually considered a black box in the ML literature, our contribution aims to open this box and gain information about the accuracy and trustworthiness of the models built downstream of this process.

The paper is structured as follows. In the “Methods”, we will define the three metrics mentioned above for the quality dimensions of representativeness, reliability, and accuracy: the *degree of correspondance*, the *degree of weighted concordance*, and the *degree of fineness*, respectively (see Table 1). In the “Results”, we will instantiate the above metrics on a realistic case of MAI, taken from the domain of MRI interpretation. In the “Discussion”, we will comment on the methods proposed in more detail, and we will also propose an interpretation of our framework within the wider perspective of Explainable Artificial Intelligence (XAI) [8]. Finally, in the “Conclusion” sections, we will outline the main contributions of this article and propose a research agenda grounded upon them.

## Methods

In what follows, we will propose novel methods to compute the scores of three quality dimensions related to the training data of machine learning AI: the accuracy of the Gold Standard and the reliability of the data from which this labelling has been derived through, respectively, the *degree of fineness* and the degree of *correspondance*; and the representativess of those data (with respect to either reference data, other training data or single instances) through the *degree of weighted concordance*.

### Reliability

The intuitive notion of reliability is straightforward: how much can we *rely upon* an agent to make decisions? Similarly (although metaphorically) we can assess how much we can rely on the data to train a predictive model with to have it make realistic predictions. Despite the broadness of this concept, which we treated in a companion article published recently [5], we here focus on the metrological interpretation [9] of reliability: this latter regards *precision* of measurements and, broadly meant, *consistency of performance*: e.g., models that give similar output for similar inputs, and raters who attach the same label to the same case. Indeed, here we focus on the even narrower technical understanding of reliability as the complement of *inter-rater variability* [10]. In this sense, assessing reliability is evaluating the degree to which the observed agreement among the raters is expected to be genuine, and not due to chance.

According to the above perspective, we can speak of reliability of a Gold Standard only in terms of the reliability of the Diamond Standard from which the former has been derived, by means of some specific reduction. In its turn, the reliability of a Diamond Standard regards the extent this set of annotations expresses a *unitary* interpretation of the single cases observed, despite the multiplicity of views entailed by the different raters involved in interpreting each case [11]. If all of the raters agree upon each and every case, then no disagreement among the case’s annotations is observed, and the reliability is maximum.

Over time, many metrics have been proposed to estimate the *inter-rater variability* (also known as inter-rater reliability and inter-rater agreement) within a dataset, like the Fleiss’s Kappa, the Cohen’s Kappa, and the Krippendorff’s Alpha [12]. These indices aim to go beyond the simple proportion of matched pairs (a score called Proportion of Agreement, and usually denoted as *P*_*o*_). This aim is motivated for the important, and often neglected, limitation of the *P*_*o*_: this score includes the amount of agreement that could be due to chance, and hence it produces an overly optimistic measure of the real agreement (and hence reliability).

All of the proposed metrics present some limitations, for instance in regard to their ability to account for missing values, or to account for ratings of different nature (e.g., categorical or ordinal), and all of them are subject to a number of paradoxes, e.g., they have been shown to behave paradoxically when the cases to be rated are not well-distributed across the rating categories [13]. Moreover, all these measure of inter-rater agreement employ a generic model of chance effects that does not take into account background information provided by the raters themselves.

To address this gap in the literature, in the recent article mentioned above [5] we proposed two novel metrics of inter-rater reliability that employ a model of chance based on the information provided by the raters: the degree of concordance (*σ*) and the degree of weighted concordance (*ϱ*). The former can be seen as the degree of *genuine* agreement among the raters, on the basis of the number of agreements and the rater’s confidence of their ratings (see also Appendix A); *ϱ* is a generalization *σ* where each single agreement is weighted by the estimated accuracy of the raters’ involved. While in this work we focus on *ϱ*, here we briefly recall the formula for both metrics (as *σ* is used in Appendix A):

1 $$\begin{array}{*{20}l}  \sigma(S, R, C) &= \frac{1}{|S|}\sum\limits_{x \in S}{m \choose 2}^{-1} \sum\limits_{r_{i} \neq r_{j} \in R} {GA}_{x}^{C}\left(r_{i}, r_{j}\right) \end{array} $$

2 $$ {\begin{aligned} \varrho(S, R, C) &= \frac{1}{|S|}\sum\limits_{x \in S}{m \choose 2}^{-1} \sum_{r_{i} \neq r_{j} \in R} {GA}_{x}^{C}\left(r_{i}, r_{j}\right)\cdot P\left(r_{i}(x), r_{j}(x) \text{ correct}\right) \end{aligned}}  $$

where *S* is the set of cases annotated by the raters; *R* is the set of raters; *C* is the |*S*|×|*R*| matrix of reported confidence degrees; *P*(*r*_*i*_(*x*),*r*_*j*_(*x*) correct) is the conditional probability (given that the two raters agreed) that the annotation provided by the raters for case x is correct; ${GA}_{x}^{C}\left (r_{i}, r_{j}\right)$ is the (chance-discounted) agreement between raters *r*_*i*_ and *r*_*j*_, defined as

3 $$ {GA}_{x}^{C}\left(r_{i}, r_{j}\right) =\left\{\begin{array}{lc} 0 & r_{i}(x) \neq r_{j}(x)\\ \hat{c}_{i}(x)\hat{c}_{j}(x) & otherwise \end{array}\right.  $$

where $\hat {c}_{i}(x)$ (resp. $\hat {c}_{j}(x)$) is the corrected confidence reported by rater *r*_*i*_ (resp. *r*_*j*_) for case x. Thus, *ϱ* can be considered as a generalization of *σ* in which the accuracy of the raters is taken into account.

For further information about the derivation of the proposed metrics and its advantages in comparison with existing approaches we refer the reader to [5]: here we discuss about how our metrics relate to a different approach for reliability assessment (mainly in the information fusion literature) based on Dempster-Shafer theory of evidence [14, 15]. While this latter theory has been widely applied to model the reliability of imprecise, uncertain and conflicting sources of information [16–19], the study of inter-rater agreement has been mainly considered under the Bayesian/probabilistic framework and, to the knowledge of the authors, the evidence-theoretic approach has not been applied to develop measures to quantify this form of reliability. As we show in Appendix A this may be due to the inability of Dempster combination rule to properly differentiate between genuine agreement and agreement due to chance (for further detail about this distinction see [5, 20]): this is the key concept in the measurement of inter-rater reliability; indeed the purpose of this dimension may be described as quantifying the amount of observed agreement that is not due to chance. Despite the incompatibility between the standard interpretation of Dempster-Shafer theory and the quantification of inter-rater reliability, in Appendix A we show how the proposed metrics can be interpreted as arising from the evidence-theoretic framework by relying on non-standard aggregation rules discussed in the literature to avoid some shortcomings of the Dempster rule of aggregation [21].

Finally, we discuss two aspects of the proposed metrics, which are related to the additional data elements that are required to compute *ϱ*. First, we note that the computation of *ϱ* requires an estimate of the accuracy of the raters. This could be obtained in multiple ways, among which:

- Through the use of standardized tests, in a sort of pre-testing certification: this is often the optimal choice but it is of difficult application;
- Employing a statistical model (such as Rasch model [22]) to estimate the raters’ accuracy: while effective, this approach may require additional information (such as the complexity of the cases);
- Equating each rater accuracy to the number of times they agree with the labeling obtained by the majority of the other raters [23];
- Equating the raters’ accuracy to the fraction of times they are in agreement with an external reference (such as the result of an existing diagnostic test).

The second aspect regards the confidence expressed by the raters in their annotations: this information is employed in the computation of *ϱ* in order to develop a model of uncertainty of the raters, so as to account for chance effects in its computation. One possible limit of this approach is that this elicitation process in itself may be affected by uncertainty: the phenomenon of *intra-rater* variability, that is the degree of self-agreement of a single rater among repeated administrations of the same test, has been widely studied and reported in the medical literature [24, 25]. Here we propose a simple but effective approach to measure this intra-rater variability, and to incorporate such an estimation into the method to compute *ϱ*. In essence, the proposed approach involves a simple modification of the data annotation process: a small random sub-sample of cases must be repeated within the annotation sequence, after an adequate interval (e.g. the repetitions may be placed after tens of new cases). Thus, each rater is asked to re-annotate some of the cases multiple times (unaware of this), and an estimate of their variability can be computed.

Specifically, let *x*^1^,...*x*^*k*^ be the repetitions of a given case *x*; *r* a rater and $c_{r}^{i}$ the confidence expressed by rater *r* on the *i*-th repetition of case *x*. Thus, denoting the standard deviation of the confidence on case *x* for rater *r* as $c_{\sigma }^{r}(x)$, the *intra-rater variability* of rater *r* on case *x* can be defined as the width of the corresponding 95% confidence interval, that is $irv(x, r) = 1.96\frac {c_{\sigma }^{r}(x)}{\sqrt {k}}$ and the average *intra-rater variability* of rater *r* can be defined as $irv(r) = \frac {1}{|X_{rep}|}{\sum }_{x \in X_{rep}} irv(x, r)$, where *X*_*rep*_ is the set of repeated cases in the annotation sequence.

Thus, how can we use this estimate of intra-rater variability in the computation of *ϱ*? First, we note that intra-rater variability *irv*(*r*) defines (for each rater *r* and case *x*) an interval-valued estimate of the rater’s reported confidence, as $\hat {c}_{r}(x) = \left [ c_{r}(x) - irv(r), c_{r}(x) + irv(r)\right ]$. Second, it is easy to observe that *ϱ* is monotone with respect to the reported confidence levels: therefore *ϱ*(*S*,*R*,*C*_∗_)≤*ϱ*(*S*,*R*,*C*^∗^) where *C*_∗_ is the matrix obtained from *C* by exchanging each *c*_*r*_(*x*) with $inf \{\hat {c}_{r}(x) \}$, and *C*^∗^ is similarly obtained by exchanging each *c*_*r*_(*x*) with $sup \left \{ \hat {c}_{r}(x) \right \}$. Therefore, by using the interval-valued confidence (which describes the elicitation uncertainty of each rater through its intra-rater variability) we can obtain a robust interval-valued estimate of *ϱ* as:

4 $$ \hat{\varrho}\left(S, R, \hat{C}\right) = \left[\varrho\left(S, R, C_{*}\right), \varrho\left(S, R, C^{*}\right)\right]  $$

We note that the original formulation of *ϱ*(*S*,*R*,*C*) can be easily obtained from $\hat {\varrho }\left (S, R, \hat {C}\right)$ as $\varrho (S, R, C) = \frac {\varrho (S, R, C_{*}) + \varrho (S, R, C^{*})}{2}$, since all interval-valued confidence levels are symmetric.

### Fineness

Intuitively, the *degree of fineness* (*Φ*_*red*_(*G*,*R*)), for a Gold Standard *G* and a set of raters *R*={*r*_1_,...,*r*_*m*_} involved in its production, is an estimate of its actual accuracy, which obviously is unknown. More technically, *Φ*_*red*_ is the *expected fraction* of instances whose labels in *G*, obtained from the Diamond Standard *D* by means of a given *reduction* (*red*), match the theoretically correct (but not necessarily known) labels. In this section we will how the *degree of fineness* varies with different reductions and how it can be computed for some noteworthy reductions. In Appendix B, we will discuss the relationship between the *degree of fineness* and computational learning theory, showing how this degree has an impact on the capability of any algorithm to learn the correct mapping between instances and labels.

Let then *R*={*r*_1_,...,*r*_*m*_} be *m* raters independently labeling the cases in dataset *D*; let also assume that each *r*_*i*_ has a constant error rate *η*_*i*_ and let *Y*={*y*_0_,...,*y*_*n*_} be the set of possible class labels. For a given case *x* let *R*(*x*)=〈*r*_1_(*x*),...,*r*_*m*_(*x*)〉 be the vector of class assignments and *R*(*y*)={*r*_*j*_∈*R*|*r*_*j*_(*x*)=*y*∈*Y*} be the set of raters that assigned label *y* to case *x*.

A reduction [26] is a function $red : Y^{m} \mapsto \mathcal {C}(Y)$, that is a function that maps any vector of class assignments *R*(*x*) to a given *structure* over the labels: examples of $\mathcal {C}(Y)$ would be *Y* (if the reduction returns a single label, as in the case of majority voting) or the collection of probability distributions over *Y*. Different reductions have been considered and proposed in the literature [26], in this article we will focus only on a set of single-label reductions, namely the *majority reduction*, which simply considers the majority vote among the raters in *R* (that is, the mode),

5 $$ maj(x | R) = {argmax}_{y \in Y} |R(y)|  $$

and the *probabilistic reduction*, according to which each of the possible labels is associated with a probability equivalent to the proportion of raters who voted for that label, that is:

6 $$ prob(x | R) = \left\langle \frac{|R(y_{0})|}{|R|},..., \frac{|R(y_{n})|}{|R|} \right\rangle  $$

We will also consider the *confidence weighted* and *accuracy weighted* reductions, which are simply defined as weighted versions of the majority reductions:

7 $$\begin{array}{*{20}l} conf(x | R) &= {argmax}_{y \in Y}\sum\limits_{r \in R : r(x) = y} {conf}_{r}(x) \end{array} $$

8 $$\begin{array}{*{20}l} acc(x | R) &= {argmax}_{y \in Y}\sum\limits_{r \in R : r(x) = y} 1 - \hat{\eta}_{r} \end{array} $$

where *conf*_*r*_(*x*) is the degree of confidence reported by rater *r* for its annotation of case *x*, and $\hat {\eta }_{r}$ is an estimate of the accuracy of rater *r*.

How can we compute the *degree of fineness* *fineness*_*red*_ for a given reduction? As we mentioned, this is defined as the probability that the labels obtained by means of *red* are actually correct. This is the inverse of the probability of error for *red*, that is

9 $$ {fineness}_{{red}}(G, R) = \frac{1}{|G|} \sum\limits_{x \in G} 1 - P_{{red}}(error)  $$

where *P*_*red*_(*error*)=*P*[*red*(*R*(*x*))≠*y*_*x*_], where *y*_*x*_ is the correct (in principle unknown) label associated with *x*. Thus, in order to determine the formula of *Φ*_*red*_ for a given reduction *red*, it suffices to quantify the probability that this reduction commits an error on an arbitrary case *x*.

Consider the case of the majority reduction *maj*(*x*). Then *P*_*maj*_(*error*) amounts to the probability that at least $\frac {m+1}{2}$^2^ raters made a classification mistake, this probability can be computed via the *Poisson binomial distribution*:

10 $$  P_{maj}(error) = \sum\limits_{k=\frac{m+1}{2}}^{m}\sum\limits_{A \in F_{k}}\Pi_{i \in A}\eta_{i}\Pi_{j \notin A}\left(1 - \eta_{j}\right)  $$

where *F*_*k*_ is the family of sets in which exactly *k* observers gave the wrong labeling. Via the Chernoff bound, and omitting some terms, we can upper bound *P*(*error*) as:

11 $$  P_{maj}(error) \leq e^{-\frac{m+1}{2}log\frac{m+1}{2\mu}}  $$

where $\mu = \sum _{i} \eta _{i}$. Thus, it is easy to observe that the *degree of fineness* of the majority reduction increases exponentially with both *increasing number of raters* and *their accuracies* (i.e. 1−*η*_*i*_).

We now consider the analogous result for the probabilistic reduction. In this case, without loss of generality, let *Y*={0,1} and let for case *x*, *prob*(*R*(*x*))=〈*p*_0_,*p*_1_〉. We can quantify the error probability *P*_*prob*_(*error*) by applying a re-sampling argument [27]: that is, we sample for case *x* a new label (which is then used as the label for *x*) according to distribution *prob*(*R*(*x*)). What is then the probability that an extracted sample would have the wrong label? This probability is given by:

12 $$  \begin{aligned} P_{prob}^{1}(err)& = p_{0}* {m \choose m_{0}}\eta_{R}^{m_{0}}\left(1- \eta_{R}\right)^{m - m_{0}} \\&+ p_{1} {m \choose m_{1}}\eta_{R}^{m_{1}}\left(1- \eta_{R}\right)^{m - m_{1}} \end{aligned}  $$

Notice that in general $P_{prob}^{1}(err) > P_{maj}(err)$: nevertheless, the main advantage of the probabilistic reduction is that we do not discard the minority labels (this is especially significant when the margin of majority is small). It is also easy to show that the error rate of the majority reduction can however be approximated in the asymptotic case through a re-sampling strategy: namely, we can sample multiple times the distribution for case *x* and then take the majority reduction of the new labels sampled via the probabilistic reduction. Given a number *k* of re-samples we can define the probability of error as:

13 $$ P_{prob}^{k}(err) = \sum\limits_{i=\frac{k+1}{2}}^{k}{k \choose i}P_{prob}^{1}(err)^{i}\left(1 - P_{prob}^{1}(err)\right)^{k - i}  $$

Then the following result holds:

#### **Proposition 1**

∀*η*_*R*_,*ε*∈(0,1) ∃*k* such that $\left |P_{prob}^{k}(err) - P_{maj}(err)\right | < \epsilon $.

While we do not directly present an analytical form for the *degree of fineness* of the confidence and accuracy weighted reductions, it is easy to observe that if $\hat {\eta }_{r}$ (resp. *conf*_*r*_) are co-monotone with *η*_*r*_ then the *degree of fineness* of this two reductions is greater than the one for the majority reduction.

### Representativeness

The quality of the data used to train a ML model is a multifaceted concept. The *degrees of concordance and fineness* are scores conceived to quantify this quality from the perspective of accuracy in conditions of uncertainty due to the lack of a true reference (this is why we also need to evaluate the reliability of the original data). A third component of data quality, no less important than the other two, is representativeness, especially in light of the need to have a decision support of real utility in real-world practice. Intuitively, “representative” is a term that equally applies to individuals, with respect to a group from which they are ideally drawn; and to groups, with respect to wider groups, or populations, from which these groups are drawn as samples. Therefore, we will focus on *representativeness* in terms of 1) the degree to which a new case that has not been drawn from the training data is nevertheless represented in this latter data (we can also speak of *compatibility* between the former and the latter, see Fig. 1); and 2) the degree to which the training data is representative of the reference population; or for the sake of availability, of a proxy set randomly drawn from this population.

Both above uses require the comparison of the distributions of two populations: this is a topic that has been widely studied in statistics literature. Indeed, there exist two main approaches for comparing (general, that is both uni-variate and multi-variate) distributions [28]: *integral probability metrics* and *divergences*. The first approach has been originally proposed in the statistical literature to prove convergence theorems for distributions: indeed, most non-parametric tests for distribution equality can be described in terms of integral probability metrics (e.g. Kolmogorov Smirnov test [29], or Maximum Mean Discrepancy test [30]). On the other hand, the second approach is based on information-theoretic divergences (e.g. the Kullback-Leibler and Renyi divergences [31]) or metrics (e.g. Jensen-Shannon distance [32]). While these two approaches have the same goal of assessing whether two distributions are similar, they have different properties:

- Integral probability metrics based approaches are better suited at comparing continuous distributions; are completely non-parametric (they require only the empirical distribution functions, edf); and, at least in the univariate case, they can be estimated efficiently and effectively [28];
- Divergences, on the other hand are better suited at comparing discrete distributions (estimation of divergences for continuous distributions may either require using the edf to fit a given parametric form, binning of the edf, or employing non-parametric estimation methods that however have limited convergence guarantees [33]), and their estimation is, in general, harder computationally [34].

Thus, while methods based on integral probability metrics have been considered more effective [28] for distribution equality testing, divergences are comparatively more effective and powerful (in the statistical sense) for the implementation of goodness of fit tests [35] (i.e., a specific form of distribution equality test where the reference distribution is in a parametric family).

Focusing on how *representativeness* can be computed, the simplest way is to consider, when available, the reference distributions of the single features: this is the so-called *univariate representativeness*. To this aim, multiple tests can be applied: when the features are categorical, the *χ*^2^ test or the *G test* [36] can be used. On the other hand, if the features are ordinal or continuous, non-parametric tests (like the *Kolmogorov–Smirnov* test) can be applied. Also divergences can be employed for this purpose: in this case the *p*-value of the test must be computed through a bootstrap or permutation procedure, as the distribution of these measures in the general non-parametric setting is not known. In all these cases, the *p*-value represents a degree of the extent the distribution of each feature can be considered as similarly shaped with respect to the (corresponding features in the) reference population. This approach rests on the strong assumption that the features are mutually independent, and requires strategies to aggregate the representativeness degrees of each of the features: this could be done by applying an aggregation function [37] (e.g. the mean, or a t-norm) to the obtained *p*-values.

A more sound approach would rather use the full joint distributions for the two populations under comparison: this approach defines a *multivariate representativeness*. This kind of representativeness represents the similarity between the two distributions more accurately than the uni-variate approach, as it allows taking the interaction among the features in due consideration. For this purpose, one could apply the multivariate versions of the corresponding statistical tests (e.g., see [38] for a multivariate extension of the Kolmogorov–Smirnov test, or [39] for a test based on order ranks comparison). A major limit of these tests is that they can be considered computationally feasible only when the number of features is relatively low (e.g. 2 or 3): the cost for computing the relevant statistics scales poorly with the number of the features or instances. Tests based on kernels have been proposed to avoid this limitation (e.g. the Maximum Mean Discrepancy method [30]): however, these require to specify an appropriate kernel function and their power have been shown to scale poorly with respect to dimensionality [40]. Similar limits also affect tests based on divergences, for which, as previously discussed [28], obtaining high-quality estimators may be difficult, especially so in high dimensional contexts [41, 42].

Thus, to consider the full dimensionality of the dataset, and avoid its “curse”, we propose a novel metrics: the *degree of correspondance* (*Ψ*). The main idea is to match each data point in the smaller dataset with the most similar ones in the larger dataset to substitute these latter points with those from the former set. Then, we assess if this substitution has changed the topology of the resulting population. This approach is similar to comparing the topology of the two considered groups with the Maximum Mean Discrepancy approach [30]. Compared with this latter approach, however, our solution does not consider the entire distributions but only the points that can be considered to be more important for the comparison: for this reason, our approach can be considered as less sensitive to outliers (i.e., to instances belonging to low-probability regions).

More precisely, the method to compute the *Ψ* is the following one:

1. Compute the distance distribution in the larger group (e.g., reference population);
2. Match each instance in the smaller sample (e.g. the training set) with the most similar instance in the larger sample;
3. Then substitute the instance in the larger dataset and compute the deviation *δ* between the pre- and post-substitution distributions;
4. Perform a bootstrap procedure to compute the approximate distribution of deviations and compute the *p*-value of *δ*;
5. The obtained *p*-value is *Ψ*. The degree of correspondence is then the probability that the two datasets (or more diverse ones) come from the same population.

In Appendix C, we provide more details on the algorithm described above.

### Data collection and illustrative experimentation

In order to provide an example of application and illustrate (through a formative example) the proposed ideas, we devised a realistic user study in which we involved 13 radiologists from the IRCCS Orthopedics Institute Galeazzi of Milan (one of the largest Italian research hospitals specialized in musculoskeletal disorders).

In this user study, we asked each of the 13 raters to separately and independently annotate a sample of 417 cases randomly extracted from the Stanford MRNet dataset [43]. This dataset, by the Stanford University School of Medicine, encompasses 1,370 knee MRI exams performed at the Stanford University Medical Center: in particular 1104 exams are abnormal, of which 319 Anterior Cruciate Ligament (ACL) tears and 508 meniscal tears. Among these cases, only the normal cases and those cases with either ACL or meniscal tears were considered: specifically, the raters were asked to establish the MRNet cases that were positive, and indicate whether these regarded either ACL or meniscal tears. More in detail, the raters had to say whether the presented imaging presented a case of ACL tear (yes/no), or a meniscal tear (yes/no): hence two classification decisions in total.

The radiologists were also requested to assess each own rating (of each case) in terms of the confidence with which they classified the case, on an ordinal scale. Our sample was balanced in order to respect the distributions of abnormal cases and type of abnormality in the original MRNet dataset.

The labels provided in the MRNet dataset were considered as the correct labels associated to the cases. This allowed us to compute the raters’ accuracy in two ways: by comparing the performance of the raters with the above reference; and by comparing the performance of each rater with the majority rating of the others. The first method is obviously more precise (as its result could be thought of the *actual* accuracy of the raters) but it is also unfeasible in most real cases for the unavailability of such a reference. As we will see, the second method yielded an overstimation of the raters’ accuracy, which nevertheless was not significant (see Table 2).

**Table 2:** Estimates of the average accuracy of the raters with two different methods: comparison with the MRNet reference (which can be thought of as the raters’ “actual” accuracy); and comparison with the majority of the (other) raters. The difference between the two methods was not statistically significant at *α*=0.05 (*p*=0.68) according to a *χ*^2^ test for proportions

Estimation method	Rater’s Average Accuracy (95% CI)
Actual accuracy	0.81 [0.80, 0.82]
Accuracy wrt majority	0.87 [0.86, 0.88]

In order to evaluate the intra-rater variability of the raters we selected 2 cases from the 417 in the annotation sequence among thse that had been considered of at least medium complexity by the radiologist author: for each of these 2 cases, 5 copies were randomly inserted into the annotation sequence. Thus, the dataset annotated by the raters contained a total of 427 images.

First, we evaluated the representativeness of the random sample we extracted from the MRNet dataset with the dataset of the other MRNet instances, both via the univariate representativeness, using the Kolmogorov-Smirnov test, and the multivariate representativeness, using the *degree of correspondance* *Ψ* (based on the Kolmogorov-Smirnov and Jensen-Shannon distance, and the Maximum Mean Discrepancy) and, for comparison purposes, a non-parametric test based on the Kullback-Leibler distance (using the estimation procedure described in [33]) and the Maximum Mean Discrepancy test [30]. In order to compute all representativeness metrics, we first transformed the original images into 10-dimensional vectors via Principal Component Analysis (PCA) and then performed the statistical tests on this vector-valued transformed representation.

Second, we evaluated the reliability of the Diamond Standard: specifically, we computed the value of *ϱ*, *k* and *α* for the entire dataset. For all possible groups of size 1, 3,..., 11 raters (among the 13 respondents) we also computed the average value of *ϱ*, the average value of the *Φ* (for all the discussed reductions) and the average Gold Standard actual accuracy (i.e. the accuracy with respect to the reference labels in the MRNet dataset) for all the discussed reductions. In doing so, we could assess the relationship between these 3 dimensions.

## Results

**Fig. 2:**
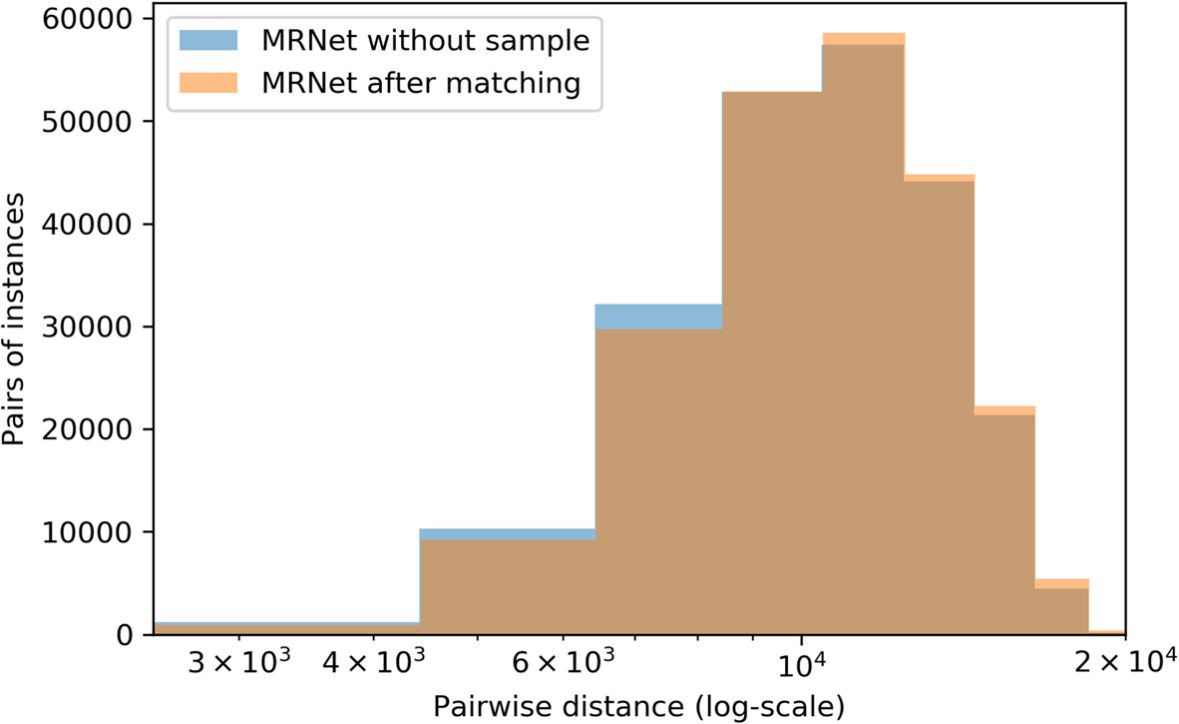
Histograms of the distribution of distances in the original MRNet dataset (after re-moving the instances in the sample we randomly selected) and after matching and substitution with the selected samples

In regard to the univariate representativeness, we found an average *p*-value of 0.50 (minimum: 0.11, maximum: 0.97) across all the features. By contrast, as regards the multivariate representativeness, the Kullback-Leibler test reported a *p*-value of 0.66, the Maximum Mean Discrepancy reported a *p*-value of 0.79. For the *degree of correspondance* (based on Kolmogorov-Smirnov, Jensen-Shannon and Maximum Mean Discrepancy, respectively) we found a *Ψ* of, respectively, 0.87, 0.74 and 1.00. The histograms of the distribution of distances in the original MRNet dataset (after removing the instances in the sample that we randomly selected) and after matching and substituting the selected samples are shown in Fig. 2: as the two distributions are almost equivalent^3^ a good metric of representativeness is expected to have a high value, close to 1. On the other hand, the large difference between the univariate and multivariate metrics of representativeness can be explained by observing that the latter ones directly rely on the topology of the data: thus they are capable to account for the correlations and dependencies existing among the features; conversely, the uni-variate representativeness assumes all features to be mutually independent and cannot quantify the large similarity between the two datasets correctly. Moreover, we note that the *degree of correspondance* *Ψ*, when based on integral probability metrics, yielded the highest similarity values, thus showing the greater efficacy of the proposed method.

**Fig. 3:**
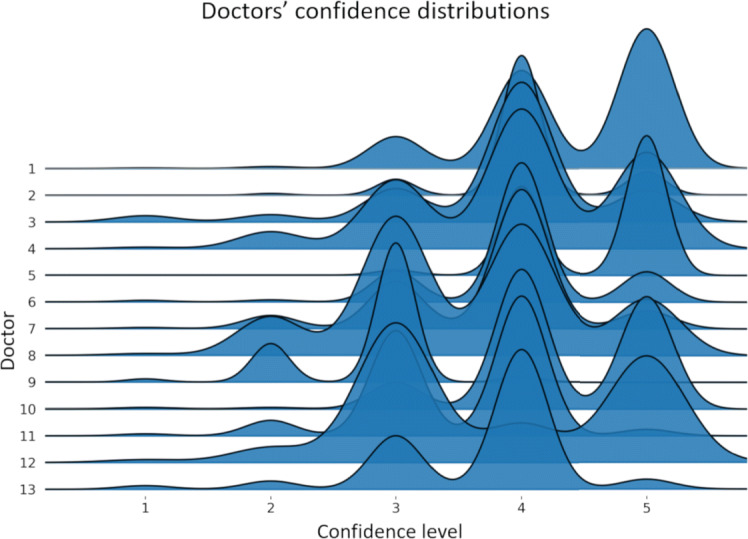
Joyplot of the perceived confidence levels reported by the 13 radiologists over the considered dataset. Each curve represents the distribution of perceived confidence levels reported by a specific annotator

The average actual accuracy of the raters was $\hat {acc} = 0.81 \pm 0.04$ (95% confidence interval) computed with respect to the MRNet reference. The distribution of the confidence levels reported by each radiologist is shown in Fig. 3. The average intra-rater variability was $\hat {irv} = 0.08 \pm 0.02$ (95% confidence interval): this means that the raters were consistent in the reported confidence degrees. For this reason, the average accuracy computed by majority was higher, but not significantly so (see Table 2).

The interval-valued $\hat {\varrho }$ between the 13 radiologists was [0.51,0.64], with average (i.e., *ϱ*) 0.57. As regards the values of Krippendorff’s *α* and Fleiss’ *k*, they were both equal to 0.63, while the value of *P*_*o*_ was 0.82. As said above, *P*_*o*_ is much higher than the other metrics because it computes simple agreement, without discounting chance effects. The relative difference between *ϱ* and the other chance-adjusted metrics is due to the different model of chance we conceived for our proposal. As already discussed in [5], our model of chance employs the confidence scores reported by the raters: in the decision-theoretic model, the underlying inter-rater reliability agreements due to chance are modeled as the probability that each rater makes a random guess (rather than an informed decision): in fact, asking raters for their confidence can be seen as a user-centered approach to estimate these probabilities. By contrast, *α* and *k* metrics do not rely on the raters’ perceptions to determine whether these latter ones had proposed an informed labelling or just guessed it; thus these metrics must resort to estimating the effect of chance from the observed distribution of annotations, which could be, in some cases, misleading.

**Fig. 4:**
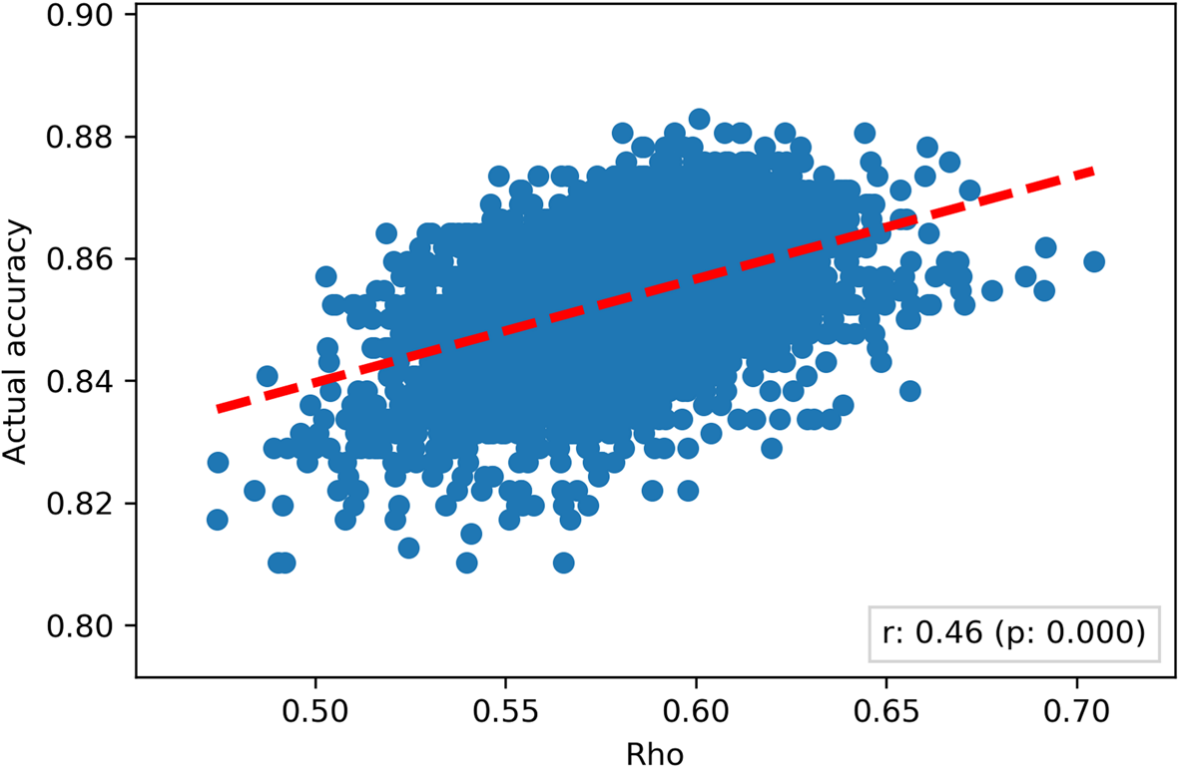
Scatterplot representing the degree of weighted concordance of each possible group of each possible size against the actual accuracy of the group. The red line represents the linear correlation between the two dimensions. The two dimensions were correlated (Pearson *r*=0.46) and the correlation was statistically significant at *α*=0.05 confidence level (*p*-value < 0.001)

The relationship between *ϱ* and the actual accuracy of the Gold Standard obtained by means of the majority reduction (for all possible group sizes) is shown in Fig. 4. The correlation between the two dimension was moderate (Pearson *r*=0.46) and statistically significant (*p*<0.001): as already discussed in [5], this means that the value of *ϱ* for a given Diamond Standard can be used as a preliminary estimate of the actual accuracy of the Gold Standard obtained from the former set: this was expected, as the *ϱ*, differently from other inter-rater reliability metrics, encompasses the raters’ estimated accuracy in its formulation.

**Fig. 5:**
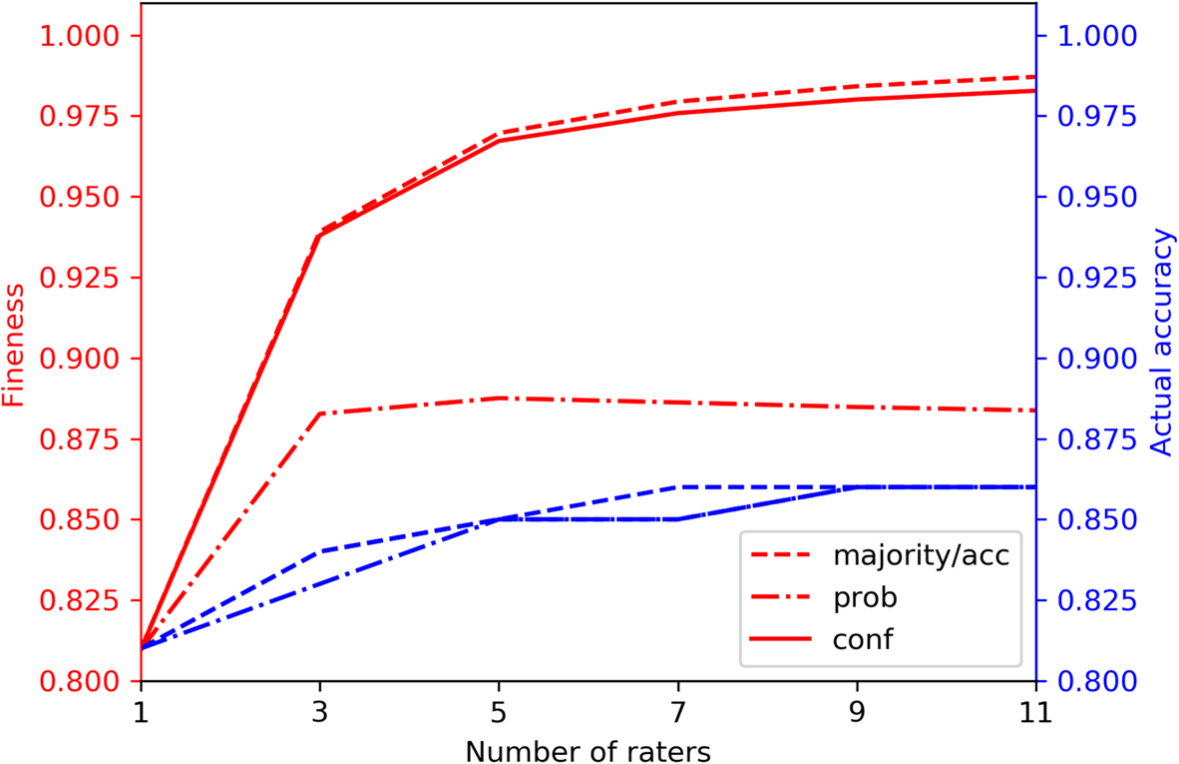
Diagram of how actual accuracy of the Gold Standard (blue lines, right scale) and its *degree of fineness* (red lines, left scale), obtained by means of the different reductions, vary with the number of raters (from 1 to 11)

The *degree of fineness* and actual accuracy of the Gold Standard (obtained by means of the different reductions), for all possible group sizes, is depicted in Fig. 5. We can observe that the estimates of accuracy provided by the *degree of fineness* metric systematically over-estimate the “actual” accuracy. This rests on the observation that the assumptions underlying the *degree of fineness* are seldom satisfied in real settings: i.e., constant and independent raters’ accuracy. In our study (and, arguably, in most real-world situations) these assumptions do not hold, especially in light of the obvious fact that doctors fail to correctly label complex and difficult cases more often than the simple ones (almost by definition).

**Fig. 6:**
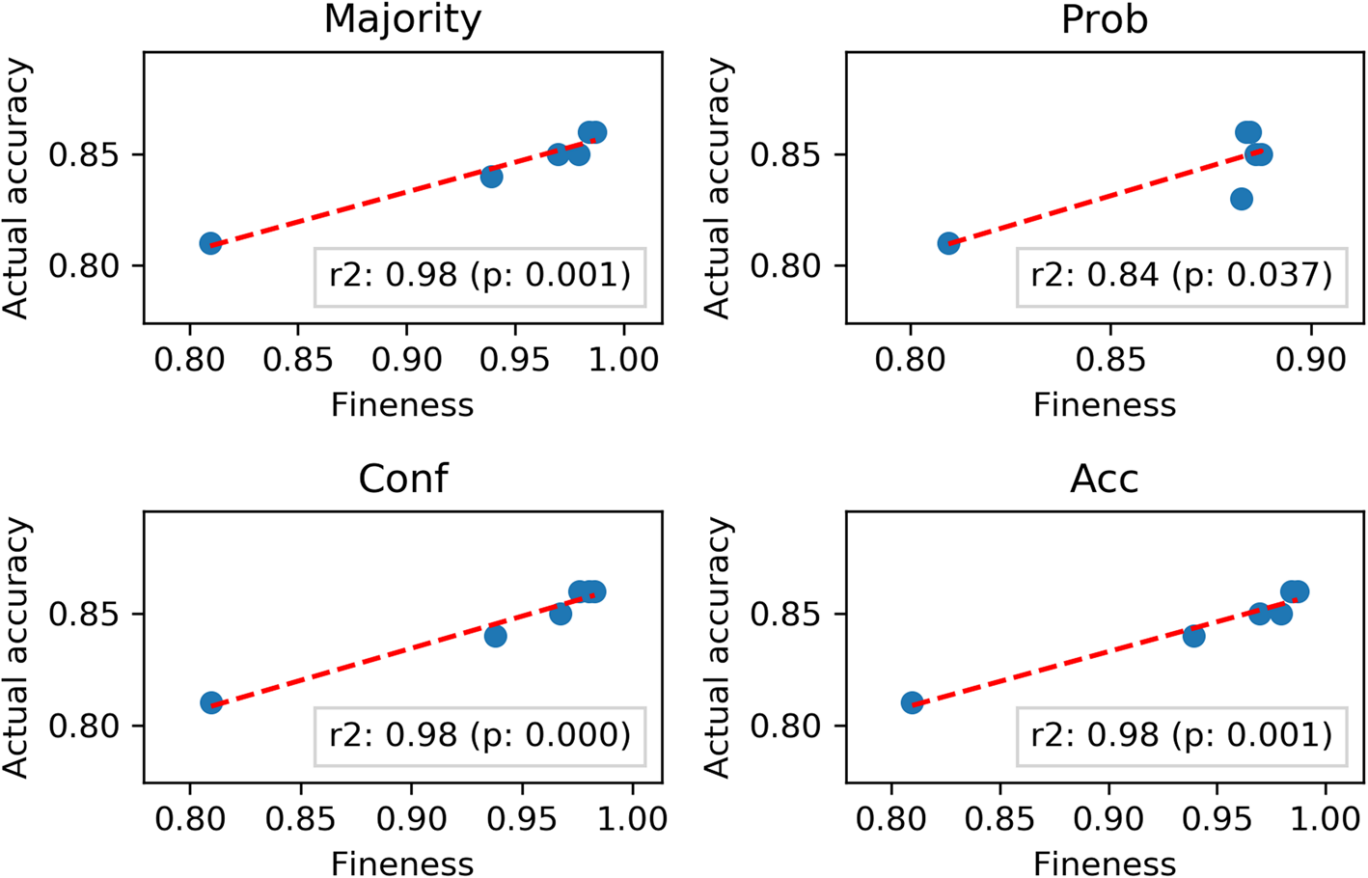
Scatterplot depicting the relationship between the Gold Standard actual accuracy and the *degree of fineness* when varying the number of involved raters (*k*) and the reduction employed. For each group size *k*, the average Gold Standard actual accuracy among all groups of size *k* was computed. For each reduction, the red line represents the linear correlation between the two dimensions: for all reductions, the two dimensions were strongly correlated and the correlation was statistically significant at *α*=0.05

The relationship between the *degree of fineness* and actual accuracy (for all considered reductions and group sizes) is shown in Fig. 6: for all of the reductions, the correlation was high (*r*>0.80) and statistically significant (confidence level 95%).

As highlighted by this correlation, we note that the *degree of fineness* can thus be considered as an adequate upper bound for, and proxy of, the Gold Standard accuracy: the two quantities are significantly correlated for all the considered reductions. Although this strong correlation may ultimately rest on the availability of precise accuracy estimates for the raters, the highlighted relationship shows that the *degree of fineness* can be seen as expressing an optimistic upper bound on the true accuracy of the Gold Standard labels. This means that, in the general case, we should expect the actual accuracy of the Gold Standard to be no higher than its *degree of fineness*, and presumably lower: suffice it to see that, if the raters’ errors were strongly correlated, the resulting accuracy would be significantly lower than the one obtained by assuming their independence.

**Fig. 7:**
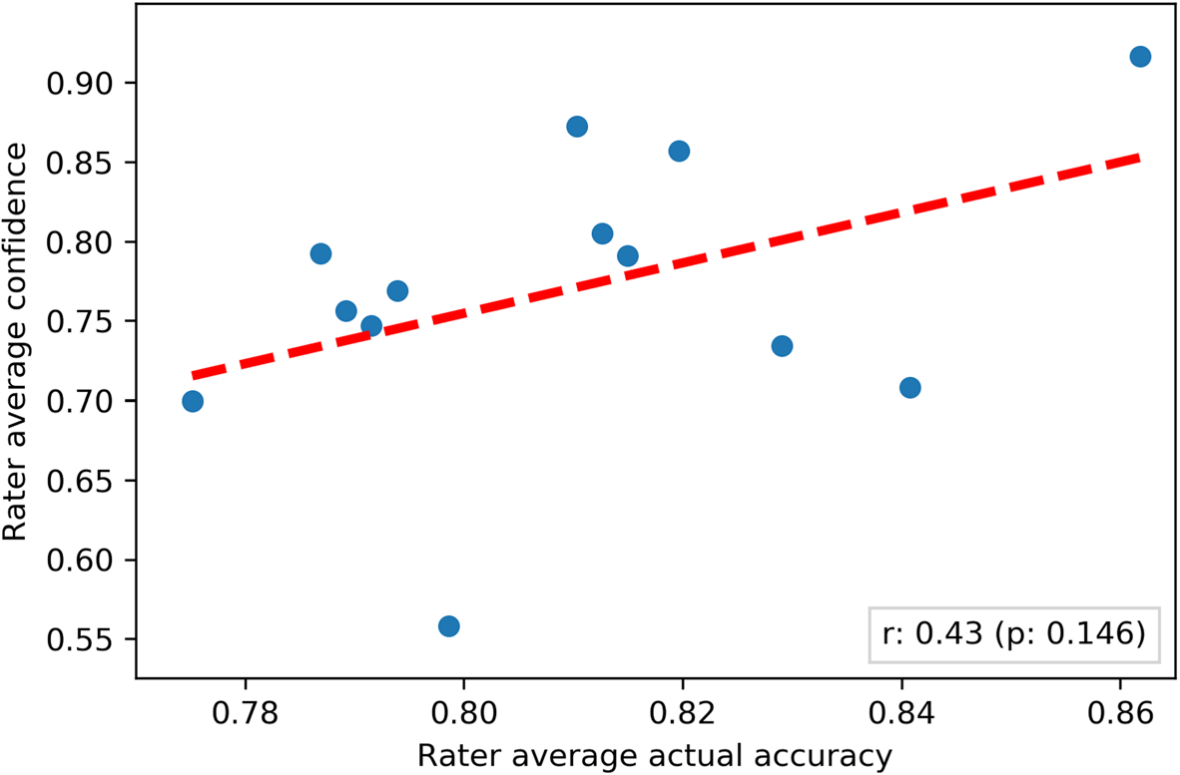
Scatterplot depicting the relationship between the actual accuracy and the confidence of the 13 raters involved in user study. The red line represents the linear correlation line between the two dimensions: the correlation was moderate (Pearson *r*=0.43) and not statistically significant (*p*=0.15), likely due to the confidence outlier at the bottom of the figure

We did not find any statistically significant difference (confidence level 95%), in terms of actual accuracy, among the different reductions, for any group size (see the blue curves in Fig. 5). To this respect, we note that all reductions were implemented so as to provide a single-valued output rather than more complex structures, like a probability distribution over labels for the probabilistic reduction. Interestingly, the performance of the accuracy-weighted and confidence-weighted reductions were very similar (and also similar to the majority reduction): this highlights a potential correlation between the raters’ accuracy and perceived confidence: indeed, as shown in Fig. 7, we found the two dimensions to be moderately correlated, although the correlation was not statistically significant. This also suggests the fact that, on most cases, confidence and accuracy levels were not so dispersed to have the related weighted reductions relevantly change the result of the majority voting.

**Fig. 8:**
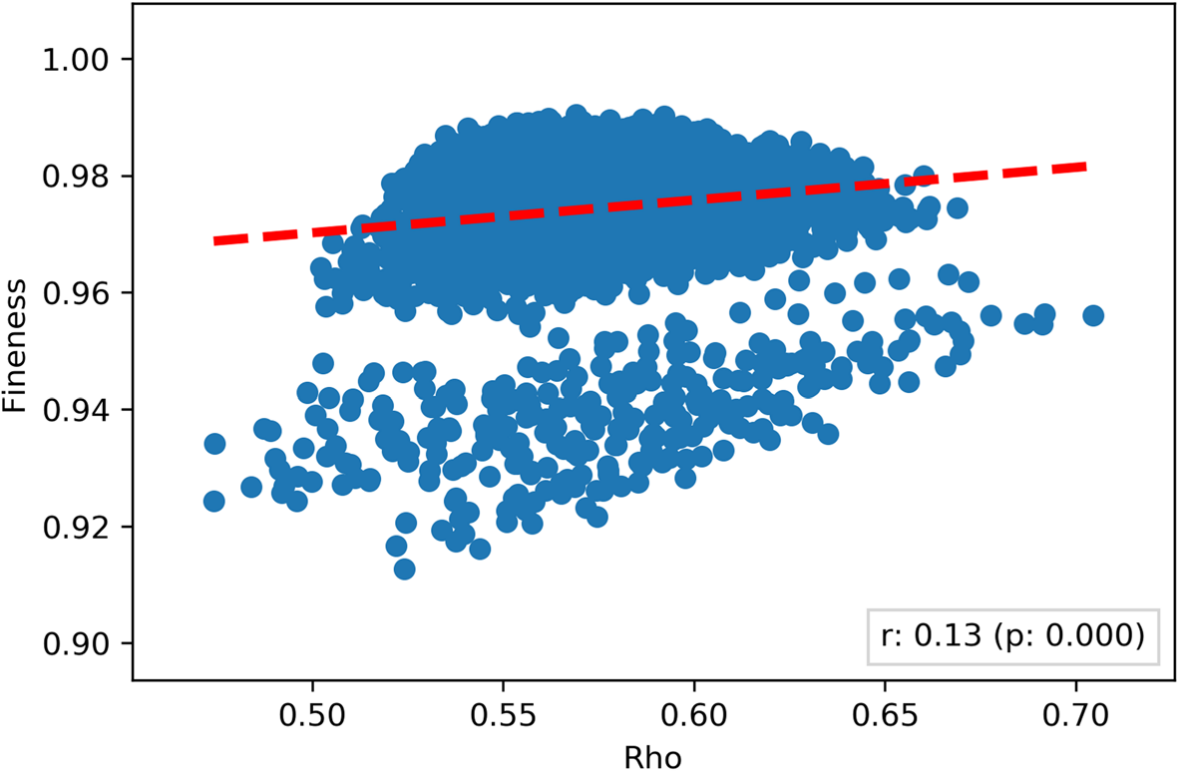
A scatterplot representing, for each possible group of each possible size, the degree of weighted concordance against the *degree of fineness* for the given group. The red line represents the linear correlation line among the two dimensions: as can be argued from the Figure, the two dimensions were weakly correlated (Pearson *r*=0.13) and the correlation was statistically significant at *α*=0.05 confidence level (*p*-value < 0.001)

Finally, the relationship between the *degree of fineness* (for the majority reduction) and the *ϱ* is depicted in Fig. 8. The correlation between the two metrics was weak (Pearson *r*=0.13) but statistically significant (*p*-value <0.001). This correlation can find an analytical justification through Eq. 10, and therefore it can be generalized: whenever the involved raters are significantly better than random raters (as it is practically always the case in professional domains), a high level of reliability is associated with a high likelihood that the *degree of fineness* of their collective effort is correspondingly high.^4^

## Discussion

In this section, we make some further comments on the results obtained in our illustrative user study, and we will discuss how these metrics can be used in practice, also beyond the applications illustrated in the previous sections.

In regard to **representativeness**, and hence the *degree of correspondance* *Ψ*, we note that this score could be used also beyond the mere assessment of the quality of a training set (with respect to the reference population). Here we mention four further possible applications. First, as hinted above, the value of *Ψ* could also be used to compute the similarity between a single data point and a given dataset: for instance, it can be used to assess the *compatibility* between a new instance to be classified by the ML model, and the model’s training set^5^. As said in the “Background” section, we can see this compatibility as a particular case of representativeness to be measured it in terms of *degree of correspondance*: first we substitute the new case in the training set; and then we compute the deviation between the original training set and the set obtained by this substitution to get the corresponding *Ψ* score. In general, low *Ψ* values for some new object to classify would warn the decision maker that any classification proposed by the model for that object should be taken with a grain of salt, as the object is different from any known object, that is from any object in the *experience* of the model.

Second: the *Ψ* score can be used to select a test set that is maximally different (i.e., for which the *Ψ* value is minimized) from the training set of the model. In doing so, the testing procedure would yield an estimate of the lower bound of the performance of the ML model on unobserved objects: estimating the prospective performance in the most conservative way could give the decision makers a way to assess the generalization capability of the model.

**Fig. 9:**
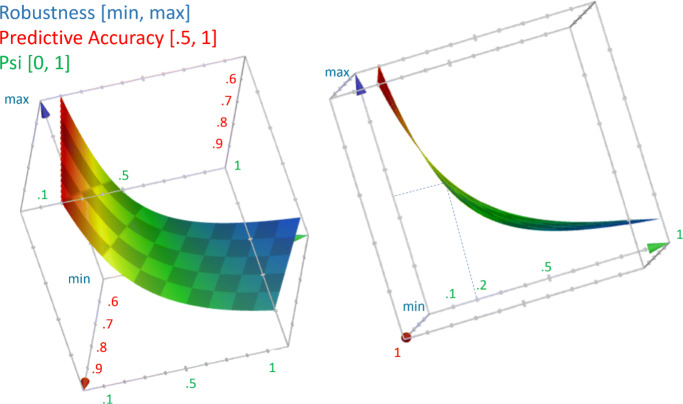
A 3D space of the model predictive accuracy (x), the degree of correspondence *ψ* (y), and the robustness of the model performance (z). 3D plot generated with GeoGebra (v. 6.0.597) http: //www.geogebra.org

Moreover, a naive but effective measure of the model *robustness*, that is reproducibility of performance in different settings from the development one, could be the ratio between the model accuracy (anyhow computed on a test set) and *Ψ*: by looking at Fig. 9, it can be noted that high accuracy scores are not associated with high robustness (qualitatively), unless the representativeness between a sample of new cases (from a different setting) and the original test set is low, at least lower than 0.2 as a rule of thumb, and very high for values below the traditional threshold of 5%.

Finally, but related to the previous uses, we propose to compute the *degree of correspondance* between the training set of a ML model and a sufficiently large dataset of cases that have been treated after putting in operation the MAI support and for which the involved doctors received an advice from the computational support. With the same features considered, if the observed *Ψ* is lower than a given threshold (like above), this could be an indication that either the population of cases is significantly changed over time; or, alternatively, that the MAI support is inducing potential *confounding medical interventions* [44]: these are interventions that would not be performed (or not so frequently) without the MAI advice, and that can significantly change the outcome of the cases considered [45].^6^ This could suggest an update of the MAI, by training its models on more current cases, or even for an update of the data schema, where at least a Boolean attribute should be added to take into consideration whether the patient received a treatment after the MAI advice or not. This case sheds light on how the assessment of the representativeness of the available data with respect to the reference target population can support *DevOps* practices of MAI maintenance, so as to contribute to high quality performance over time [46].

In regard to the dimension of **reliability**, an important point regards how the relatively low value that we observed for our Diamond Standard should be interpreted: specifically, one could wonder whether a reliability of just 0.57 for a Diamond Standard, as expressed by the *ϱ*, should be considered sufficient to produce a high-quality Gold Standards. This question is not different from wondering whether an *α* or *κ* of 0.63 is a sign of sufficient “true agreement” (besides the amount due to chance) to consider the resulting Gold Standard reliable. In this paper, we will not contribute to the long debate that has been conducted for more than 40 years, both in the statistical and medical literature, on what thresholds should be adopted to address this aspect in merely quantitative terms [5, 47, 48]. That notwithstanding, a general rule of thumb would demand that, in critical domains like medicine is, raters involved in the construction of a reliable Gold Standard should truly agree in at least two-thirds of cases; our *degree of weighted concordance*, since it is a case-wise average, can allow to check whether the lower bound of the confidence intervals of such an estimate are above this minimum requirement. Thus, rather than giving one-fits-all criteria about reliability, we emphasize the striking fact that the chance-adjusted reliability of the Gold Standards (or, better yet, Diamond Standards) used to train medical AI is usually low, very low [49]. For instance, in [11], we reported the low agreement that multiple raters achieved in two settings from different medical specialties like cardiology (i.e., ECG reading) and spine surgery (i.e., operation reporting). In the former case, in reading 3 ECGs of medium complexity the raters involved did not achieve an *α* higher than 0.6; the surgeons called to report the same operation they participated in through a standardized form achieved an *α* lower than 0.8! To this respect yet, it is important to notice that disagreements do not usually occur because some rater is less skilled than the others, and hence for interpretation errors (due to what is called *label bias* [50]); in fact, this is seldom the case. More often, it is the *intrinsic ambiguity* of the *interpretand* phenomenon that brings raters to different, yet equally plausible, interpretations [49]. Other factors that could undermine the potential agreement between raters, and hence the reliability of the Diamond Standard (and then the accuracy of the Gold Standard), are related to differences in how the raters react to the experimental conditions in which their opinions and interpretations are collected (since the process occurs often in controlled experimental settings); and more generally, to the fact of being involved in a session out of their every-day practice. This latter phenomena is generally known as “Hawthorne effect” [51], but it is not clear whether the “awareness of being observed or involved in an experiment” affects the ratings more in terms of increasing the accuracy (up to levels that in real-world settings would not be tenable, mainly for conditions of uninterrupted concentration and focused commitment); or rather in the opposite terms of its reduction (an effect known as “laboratory effect” [52]) mainly due to lack of real motivations, engagement or just of the fear of consequences in case of errors. For this reason, the *degree of weighted concordance*, since it is defined also at instance level (that is for each single case), differently from the other metrics, could be preferable: for instance, it could be used to select those cases for which the agreement is maximum (or above a specific, conservative threshold, like the one proposed by Krippendorf for medical data of 0.8 [48]), so to train the Medical AI only on those “high-quality” instances.

**Fig. 10:**
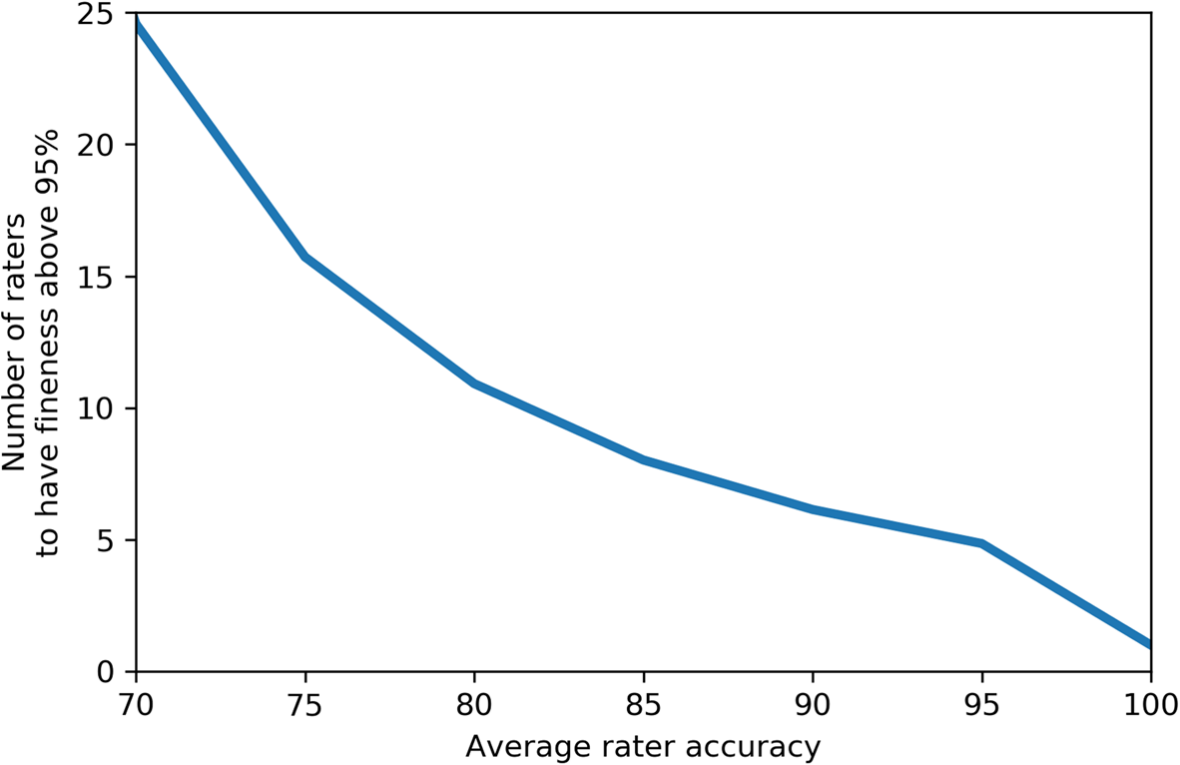
Number of raters sufficient to obtain a *degree of fineness* ≥95*%*, when varying the average accuracy of the raters. The curve is relative to a dataset of size |*S*|=427

In regard to **accuracy** and the *degree of fineness*, we noted that, even if in most cases we expect that this metric over-estimates the actual accuracy of the Gold Standard, the *degree of fineness* can still be considered as an adequate upper bound estimate for the latter dimension. In order to further comment on this aspect, in Fig. 10 we provide an analytical bound for the number of raters needed to obtain a desired level of *degree of fineness* in case of the majority reduction, by relying on Theorem 23: for instance, in our study, the above mentioned Figure shows how the number of raters necessary to obtain a “95% accurate” set of cases, when the raters’ estimated average accuracy is 81% (not too differently from the diagnostic accuracy observed in many other studies [53]), is 10. Although this number of raters to involve in annotation could seem unpractical, the reader should notice that the bound depicted in Fig. 10 should be interpreted as *optimistic*, in that it relies on the same two assumptions underlying the *degree of fineness* metric. When these assumptions are not satisfied, then the aggregated accuracy of the Gold Standard is even lower than we can analytically estimate, as shown in Fig. 5. Figures 5 and 10 also highlight that the traditional practice of relying on only 1 or, at most, 3 raters may be deeply flawed: in fact, unless we involve 100% or so accurate raters (that is, if we involve normal doctors), at least 7 raters should be involved to get a *degree of fineness* of 95%. We note that, even though these analytical bounds can provide an indication of the minimum number of raters to derive a sufficiently reliable (in the sense of “true”) Gold Standard from their ratings, nevertheless one should be wary of the likelihood of systematic errors by the raters or potential hidden stratifications [54] (e.g. by case complexity) which would have an impact on the result of the reductions performed.

With regard to the lack of significant differences between all the considered reductions, we noted that this relative similarity may be due to the fact that all reductions were implemented to provide a single-valued output while, in general, some of these reductions (e.g. the probabilistic one) can return more structured and informative output (describing the relative likelihood in favor of the different possible labels). In this light, the lack of significant differences shows that more information-preserving reductions, when restricted to return a single output, perform similarly to the majority reduction (in terms of Gold Standard accuracy), while being able to preserve more information: this latter feat has been shown to be beneficial for the training of ML models [55, 56].

For this reason, the practice of sharing not only the Gold Standard, but also the Diamond Standards, when making ML datasets available for the reproducibility and external validation of ML models, should be adopted more widely. This would allow for the selection of the most appropriate reduction for the task at hand, similarly to how model selection is usually performed.

With respect to the correlation between the *degree of weighted concordance* and the *degree of fineness*, this observation allows us to show that the two dimensions they intend to quantify are correlated (as they both relate to the quality of the Diamond and Gold Standard) but orthogonal, in that they focus on different aspects: thus, we can highlight the importance of properly taking in consideration the reliability of a given Diamond Standard, as this latter evaluation could also be used as a proxy of the quality (i.e., accuracy) of the derived (or better yet “reduced”) Gold Standard, and it hence affects all of the subsequent analyses and considerations.

Finally, we comment on the fact that, as indicated in Table 1, to compute the *degree of weighted concordance* and *the degree of fineness*, additional data are necessary, with respect to other common metrics, like the more common reliability scores. In particular, for *ϱ* we need a small additional effort by the raters involved, who are required to also express the degree of confidence with which they associate each case with the intended label. We deem this effort of small entity with respect to the advantage it yields from our personal experience: in our study we collected this information by means of a semantic differential scale, that is with a 6-value scale where only the extremes were explicitly stated, from “100% certain”, down to “definitely not sure”. Obviously, many other scales can be adopted to this aim, as the confidence scores are then to be normalized into a number from 0 to 1. To this respect, the reporting variability should be properly taken in consideration: in our study we did this by setting up an experiment to measure the intra-rater variability, and showed how this information can be used in the computation of *ϱ*.

Moreover, for the computation of the *degree of fineness* and *degree of weighted concordance*, we need to estimate the rater accuracy. As previously anticipated, this can be done in several ways, among which we mention the following ones:

1. Through the use of standardized tests, in a sort of pre-testing certification of the raters’ proficiency: this is often the optimal choice but it is of difficult application;
2. Employing a statistical model (like the Rasch model [22]): while effective, this approach may require additional information (such as the complexity of the cases);
3. Equating each rater accuracy to the number of times they agree with the labeling obtained by the majority of the other raters[23];
4. Equating the raters’ accuracy to the fraction of times they are in agreement with an external reference (such as the result of an existing diagnostic test).

While in this article we employed the fourth method (as in our case such an external reference was available), in most real cases such as an external reference would not be available: one could wonder if the obtained results would then be significantly different, if one of the other methods would have been used. For this reason, we tested the difference, if any, between the estimates of the raters’ accuracy as produced by methods no. 3 and 4 above. The results for this comparison are reported in Table 2: specifically, we found that method 3 over-estimates the accuracy of the raters (this should be expected: if the raters are of similar expertise and training background, they would be likely be in agreement between each other) but, in any case, the difference was not significant (*p*=0.68). We thus believe that, through the application of method 3, good estimates of the raters’ accuracy can be obtained also when an external reliable reference is not available, if the raters are good experts of similar expertise, as in our case.

### Implications for the XAI research field

It is common to denote Machine Learning (ML) methods as data-driven. And rightly so: most supervised ML models can be seen as but data memorization structures (or even data compressors) that are ingeniously “biased” to forget some aspects of the data upon which they have been trained (i.e., made fitted to) so that they can be conveniently applied to instances of new data, and be used to effectively guess some relevant (target) feature that is missing from the new data [57, 58].

This stance sheds light on our approach to eXplainable AI (XAI). We intend this expression quite literally: rather than AI systems that are capable to provide human decision makers with clear explanations of their “predictions” (a useful feature we purposely don’t cover here), we intend XAI as AI systems that are “open to interpretation”, that is “scrutable” to human investigation, also in that they make elements (i.e., true propositions) available on their sources or functioning by which the human decision makers who consult its advice can be helped to *account for* their final decision to somebody else (e.g., the “data subject” mentioned in the General Data Protection Regulation of the EU - GDPR) and explain *why* they made such a decision taking into account the machine’s advice. In this mould, a XAI system takes an active stance to help humans understand why they should (or should not) trust its output.

In this specific study we focused on how to make the broad class of applications of *data-driven* MAI, or ML-based MAI, *explainable*, from the perspective of the assessment of the quality of *its training data*, and in particular of the Gold Standard upon which the MAI has been trained to give its users advice for any new input data.

In this sense, we interpret explanations as *understanding support* [59], and this latter one in the light of the critique by Kelp to both the “explanationist view” and the “manipulationist views” of understanding, that is in terms of supporting (human) understanding build a “wellconnected knowledge” [60]. According to this view, understanding how a system works does not only involve knowing a set of true propositions about the system behaviors (like in case of data about predictive accuracy and feature ranking for a given prediction), but also knowing how these propositions are interrelated, within a framework of sense-making. Our contribution is a piece of this overall framework, regarding a topic that we found to have been relatively little investigated in the specialist literature and little discussed in the scholarly communities so far.

## Conclusion

We build–and trust–ML models as if we grounded them on stone instead of sand. However, this trust is misplaced. Indeed, potential concerns in the development of ML models have been highlighted by an increasing number of research works [61, 62], indicating a troubling lack of (both theoretical and, more importantly, empirical) rigor and a potential brittleness of the claimed results (due to a widespread competition mindset and the consequent risk of overfitting). The purpose of this paper was to further explore how these and other concerns about ML relate to the quality of the information sources that are used to build such systems.

To this end, consider an ML model that, trained on a large and representative set of medical cases, achieves an accuracy of 96% on a hold-out test set. Who would be disappointed by such a model, which exhibits such a performance on a complex medical task? The answer is almost no one, and rightly so. However, we still neglect to consider whether the data experience at the basis of the model is as solid as stone or rather as crumbly as sand. For instance, if, for our model, the Gold Standard is produced by three top “board-certified” radiologists, who are 89% accurate in their diagnosis on average,^7^ the ML model would be at most 93% accurate; if the raters were average diagnosticians, the model would perform even worse: 87%. Even worse, we would still believe the model had a 96% accuracy.

Should we stop building ML models and the related MAI, as we cannot afford robust foundations, such as involving at least 9 raters for each and every Gold Standarding task? Not at all: as Borges once wrote (in *In Praise of Darkness*, 1974) “nothing is built on stone; all is built on sand, but we must build as if the sand were stone.” While data scientists use their ground truths as if they were stone, we should never forget that this is illusory, and both developers and users should become aware of the fragile nature of ML applications, and in particular, of the intrinsic limits of MAI. When we say intrinsic, we refer quite literally to the quality of internal data, not to mention the quality of the algorithm.

The first step to becoming aware of the quality of MAI requires having “the words to say it.” To accomplish this broad aim, we introduced a number of concepts related to the *ground truthing* process: the initial multi-rater representation (i.e., Diamond Standard) and the transformation from this representation to the Gold Standard (i.e., Reduction, many types of which can be conceived besides majority voting). We also provided intuitive, analytical, and operational definitions of some new quality constructs related to the above representations: a new case-wise reliability of the Diamond Standard (i.e., *degree of weighted concordance*); the dimension of representativeness of a training set with respect to its reference population, measured in terms of the *degree of correspondence*; and a new dimension applicable to the Gold Standard, on which ML models are trained to quantify its quality even when lacking a theoretically true representation (*degree of fineness*).

The dimensions listed above are intended to facilitate reflection on the broad aspects of reliability and representativeness before focusing on model technicalities and potentially misleading or partial performance metrics, such as accuracy, which only refers to the match between the AI predictions and the data taken from the Gold Standard.

Moreover, we instantiated these metrics in a realistic scenario, applying them to a reference dataset of MRI images (MRNet), which we had relabeled by 13 board-certified radiologists. In doing so, we provided proof of the feasibility of these quality scores and prepared the ground for new research aimed at understanding how these measures can support and positively inform human decision-makers in naturalistic real-life practice for the safe and effective application of MAI in clinical settings.

More concretely, we should devise interaction protocols that help us minimize the odds that the advice of MAIs will bias decision-makers. Instead, MAI should be seen as a peripheral and adjunct [63] component of a collective (mostly human) intelligence, which is not overdependent on its support for interpretative and judgemental tasks.

More importantly, since we usually *assume* that our Gold Standard is perfect, reflecting on its quality necessarily entails developing an informed *prudence* with regard to its reliability and adequacy for supporting decision-making in delicate domains where decisions can have legal effects on, or affect the health of, data subjects. Thus, our ultimate aim is to contribute to raising awareness of the impact of our assumptions, models, and representations in intensive cognitive tasks like medical diagnostic and prognostic decision-making.

## Appendices

### A Appendix: derivation of the degree of concordance based on the Dempster-Shafer theory

First, we briefly recall the definition of a *mass function*, i.e. a function *m*:2^*Ω*^↦[0,1] (where *Ω* is the space of alternatives) s.t. ${\sum }_{A \in 2^{\Omega }} m(A) = 1$, a mass function is said to be *simple* if it is of the form:

14 $$ m_{X}^{s} (A) = \left\{\begin{array}{lc} s & A = X\\ 1-s & A = \Omega \end{array}\right.  $$

The Dempster rule of combination is defined as the orthogonal sum of two mass functions, that is:

15 $$ {\begin{aligned} m_{1} \oplus m_{2}(X) = \frac{1}{\sum_{A, B : A \cap B = \emptyset} m_{1}(A)m_{2}(B)} \sum\limits_{A, B: A \cap B = X} m_{1}(A)m_{2}(B) \end{aligned}}  $$

First, we note that the decision-theoretic model underlying most inter-rater reliability metrics (see [5, 20]) can be formulated in terms of simple mass functions (rather than as 2-tiered probability distribution, as usually assumed): if rater *r* expressed label *y*_*r*_ by taking an informed decision (rather than an informed guess) with probability *c*_*r*_, then this is equivalent to assuming that the rater adopts the simple mass function $m_{y_{r}}^{c_{r}}$ where *s*=*c*_*r*_(*x*) and *X*=*r*(*x*). It is easy to observe that the Dempster rule of combination is inappropriate if we want to compute the mass attached to the event that two raters *r*_1_,*r*_2_ agreed genuinely (thus, not due chance). Indeed, $m_{y_{1}}^{c_{1}} \oplus m_{y_{2}}^{c_{2}}$ is not equal to 0 when the two raters are in disagreement and, further, when the two raters are in agreement on label *y* it happens that:

16 $$ {\begin{aligned} m_{y}^{c_{1}} \oplus m_{y}^{c_{2}}(y) &= c_{1}(y)c_{2}(y) + c_{1}(y)\left(1 - c_{2}(y)\right) \\&+ \left(1 - c_{1}(y)\right)c_{2}(y) \neq c_{1}(y)c_{2}(y), \end{aligned}}  $$

where the last term in the inequality would be the correct expression of the genuine agreement between *r*_1_,*r*_2_. Therefore, under the standard interpretation of evidence theory it is impossible to obtain a measure of agreement that properly discounts agreement due to chance. Nevertheless, we show that the proposed metrics arises from the evidence-theoretic perspective when we adopt two non-standard combination rules previously considered in the literature [21]. Specifically, show how the degree of concordance *σ* can be derived from the evidence-theoretic framework by using, instead of Dempster rule, the Dubois-Prade [64] and mixing [65] rules of combination:

17 $$\begin{array}{*{20}l} m_{1} \vee m_{2} (X) &= \sum\limits_{A,B : A \cup B = X} m_{1}(A)m_{2}(B) \end{array} $$

18 $$\begin{array}{*{20}l} \biguplus_{i} m_{i} (X) &= \frac{1}{n}\sum\limits_{i=1}^{n} m_{i}(X) \end{array} $$

Denote with $[y]_{x}^{R} = \{ r \in R : r(x) = y \}$, then it is easy to show that $\forall r_{i}, r_{j} \in \left [r_{i}(x)=y\right ]_{x}^{R}$ it holds ${GA}_{x}^{C}\left (r_{i}, r_{j}\right) = m_{y}^{c_{i}} \vee m_{y}^{c_{j}}$: thus, the Dubois-Prade combination of the mass functions corresponding to *r*_*i*_,*r*_*j*_ correctly represents the probability that the two raters agreed genuinely (not by chance). Having quantified the degree of genuine agreement among any pair of raters, how can we aggregate the evidence for genuine agreement allocated to a given *y*∈*Y*? It is easy to observe that applying Dubois-Prade on $[y]_{x}^{R}$ (for each *y*∈*Y*) would underestimate the degree of agreement: indeed, in this way we would compute the evidence for the event that *all* observed agreements were genuine, and not the expected number of such agreements which is the quantity that we want to compute. It is thus easy to show that the correct way to aggregate the mass functions of raters in $[y]_{x}^{R}$ (for each *y*) is through the mixing rule of combination, as this rule is define as computing the average of multiple mass functions:

19 $$ {GA}_{x}^{C}\left([y]_{x}^{R}\right) = \biguplus\limits_{\left(r_{i}, r_{j}\right) \in \left([y]_{x}^{R}\right)^{2} : r_{i} \neq r_{j}} m_{y}^{c_{i}} \vee m_{y}^{c_{j}},  $$

Therefore, the expression of *σ* can be obtained as the average of ${GA}_{x}^{C}\left ([y]_{x}^{R}\right)$ with respect to *y*∈*Y* and *x*∈*X*, that is:

20 $$ {}\sigma(S, R, C) = \frac{1}{|S|}\sum\limits_{x \in S} {m \choose 2}^{-1}\sum\limits_{y \in Y} {|[y]_{x}^{R})|^{2} \choose 2} {GA}_{x}^{C}\left([y]_{x}^{R}\right)  $$

Thus, *σ* (and by extension also *ϱ*, though the corresponding derivation is more involved) can be interpreted in the evidence-theoretic framework as the average genuine agreement (which, as shown previously can be understood as the expected degree of evidence attached to the event that the agreement between any two non-conflicting raters is not due to chance) over a set of completely conflicting information sources.

### B Appendix: degree of fineness and PAC learning

In this Appendix we discuss the connection between the *degree of fineness* and computational learning theory, by showing what the *degree of fineness* of a given reductions tells us about the generalization capacity) of a Machine Learning model trained on the result of such a reduction. The goal of computational learning theory is to establish, given standard assumptions about the data generation process (e.g. that instances are sampled i.i.d. from an unknown distribution), resource complexity bounds (usually, in terms of *sample size*) sufficient for a given Machine Learning algorithm to perform well after observing only a finite sample. One of the central concepts in this theory is that of PAC (probably approximately correct) learnability:

#### **Definition 1**

([66]) Let *H* be a class of models, *D* a distribution over the sample space *X*×*Y*. Then *H* is PAC learnable if, for each distribution *D*, *ε*,*δ*∈(0,1)^2^, there exists *m*_*H*_=*poly*(1/*ε*,1/*δ*) and an algorithm *A* which, when given a finite sample *S* of size ≥*m*_*H*_, returns an hypothesis *h*∈*H* s.t. with probability greater than 1−*δ* the error of *h* over *D* is smaller than *ε*.

While the standard definition of PAC learnability only applies to the case where the labels provided to the learning algorithm are correct, the setting that we consider does not satisfy this assumption as the raters, in general, are not expected to be perfect. For this purpose, we recall a well-known theorem characterizing PAC learnability in the presence of labeling errors (i.e. noisy oracles):

#### **Theorem 1**

([67]) Let *D* be a dataset labeled by a noisy oracle with error rate *η*∈[0,0.5), let *H* be a class of models with Vapnik-Chervonenkis dimension [68] *d*. Then *H* is PAC-learnable under noise *η* with sample complexity:

21 $$ \mathcal{O}\left(\frac{d\cdot log\frac{1}{\delta}}{\epsilon^{2}\left(1 - 2\eta\right)^{2}}\right)  $$

Then, it is easy to observe that, for any single-valued reduction (such as the majority, confidence- and accuracy-weighted ones) the learning problem of training a Machine Learning algorithm over the set (*G*,*R*(*G*)) is an instance of the learning with errors problem. Therefore, the following corollary of Theorem 1 evidently holds:

#### **Theorem 2**

Let *red* be a single-valued reduction with *degree of fineness* *Φ*_*red*_(*G*,*R*). Let *H* be a class of models with Vapnik-Chervonenkis dimension *d*. Then *H* is PAC-learnable under noise $\eta = 1 - \Phi _{red}(G,R) = 1 - \frac {1}{|G|}{\sum }_{x \in G} (1 - P_{red}(error)) = \frac {1}{|G|}{\sum }_{x \in G}P_{red}(error)$ with sample complexity:

22 $$ \mathcal{O}\left(\frac{d\cdot log\frac{1}{\delta}}{\epsilon^{2}\left(1 - 2\eta\right)^{2}}\right)  $$

Thus, this theorem provides a direct connection between the *degree of fineness* metrics and the complexity of the associated learning problem: specifically, as we know that the *degree of fineness* of a reduction increases (approximately at an exponential rate) with the number of involved raters, this result implies that both the sample complexity and the generalization error of a learning algorithm trained on the result of a given reduction can be bounded in terms of the number of involved raters.

With respect to this latter aspect, we note that the problem of determining an upper bound on the minimum number of raters needed to obtain a certain level of *degree of fineness* has been studied for the specific case of the majority reduction: indeed, Heinecke et al. [23] proved the following result:

#### **Theorem 3**

([23]) When employing the majority reduction, to obtain a desired level of *degree of fineness* *Φ*, for each case *x*∈*G* one should involve

23 $$  \mathcal{O}\left(\frac{log\frac{|G|}{1 - \Phi}}{\left(1 - 2\eta_{R}\right)^{2}}\right)  $$

raters, where *η*_*r*_ is the average error rate among *R*.

We note that the main limitation of the proposed approach to compute the *degree of fineness* lies in the assumptions of raters’ independence and existence, for each rater *r*, of a constant error rate *η*_*R*_. To avoid this limitation, more expressive techniques based on probabilistic graphical models [69] or Dempster-Shafer theory [16] could be used to account for potential dependencies among the raters and variable error rates (though these approaches may require a computationally complex estimation phase to determine the structure and parameters of the underlying joint distribution, and for this reason they may be unsuitable if we only need a rough estimate of Gold Standard accuracy).

### C Appendix: matching method in the computation of the degree of correspondence

In this Appendix, we provide a description of the formal method by which to match the instances in two datasets and to compute the deviation between the pre- and post-substitution distributions. In regard to the matching procedure, for each instance *P*_*i*_ in the largest set *P* and instance *G*_*j*_ in the smallest one *G*, let *d*(*i*,*j*) be the distance (according to any given metric) between *i*,*j*. We want to find a match between *P* and *G* such that each object in *G* is matched with exactly one object in *P* and its distance be minimal, i.e. we want to solve the following optimization problem:

24 $$ min \sum\limits_{i,j} x_{i,j}d\left(i,j\right) \text{ where } \left\{\begin{array}{ccc} \forall i {\sum}_{j} x_{i,j} \leq 1\\ \forall j {\sum}_{i}. x_{i,j} = 1\\ x_{i,j} \in \lbrace 0, 1 \rbrace \end{array}\right.  $$

which is an instance of the minimum cost assignment in unbalanced bipartite graphs problem [70]: this problem can be solved in polynomial (actually, log-linear) time. Then let *P*^′^ be a new dataset which is equivalent to *P* except that ∀*i*,*j*.*x*_*i*,*j*_≠0 *P*_*i*_ is exchanged with *G*_*j*_. Let *dist*(*P*) be the distribution of distances in *P* and *dist*(*P*^′^) the distribution of distances in *P*^′^.

In order to compute the deviation *δ* between *dist*(*P*) and *dist*(*P*^′^) any integral probability metric or (*symmetric*) information-theoretic divergence can be employed: nonetheless, despite the test for similarity in the proposed method being uni-variate (as we compare only the distributions of distances), we expect an implementation based on integral probability metrics to be more effective. In order to compute the value of *Ψ* (that is, the *p*-value of the computed deviation *δ*), as the test is completely non-parametric and the distribution of the statistic is not known, the latter can be approximated through a standard bootstrap procedure.

As regards the properties of this testing procedure, we first observe that the total computational cost of computing *Ψ* is quadratic: more precisely, the complexity of the procedure is dominated by the computation of the pairwise distances which requires *O*((|*P*|+|*G*|)^2^) time. Thus, the algorithm has good scaling properties with respect both the dimensionality and dataset size: we further note that, when the size of the groups is large, approximate matching algorithms [71] can be used to speed-up the computation of *Ψ* (poly-logarithmic time can be achieved using randomized or online algorithms, with guaranteed solution quality bounds). Second we note that the following desirable property holds:

#### **Theorem 4**

*Ψ*=1 iff *G*⊂*P*

#### *Proof*

The implication *G*⊂*P* ⇒ *Ψ*=1 is obvious. For the converse, simply note that for *Ψ*=1 the deviation between *dist*(*P*) and *dist*(*P*^′^) must be strictly smaller than the deviation between any two possible splits of the merged dataset. □

## Data Availability

The annotation data that support the findings regarding the machine learning experiment are property of the IRCCS Orthopedic Institute Galeazzi. Data can be shared upon reasonable request and with permission of the above Institute. The code of the scripts used in the experimental part, and an online service for the computation of the reliability score and the degree of representativeness, are available at https://github.com/AndreaCampagner/qualiMLpy/.

## Abbreviations

ACL: Anterior Cruciate Ligament
DS: Diamond Standard (dataset)
GT: Gold Standard
ICC: intraclass correlation coefficient
IID: Independent and Identically Distributed
IRCCS: Istituto di Ricovero e Cura a Carattere Scientifico
IRR: Inter-Rater Reliability
MAI: Medical Artificial Intelligence
ML: Machine Learning
MRI: Magnetic Resonance Imaging
PAC: Probably Approximately Correct
PCA: Principal Component Analysis
PRO: Patient Reported Outcome
SEM: standard error of measurement
TA: True Agreement
XAI: eXplainable AI

## Footnotes

1 In fact, in the case of decision *support*, we can assume further basic determinants of trust, as these have been conceptualized in [6]: like the trustee’s integrity, equatable to behavioral stability, which in humans is supported by referring to a stable framework of values and by following a clear set of principles and rules, and in machines it follows from their deterministically algorithmic nature; and the trustee’s benevolence, which is a more delicate matter (as this should be reflected into the manufacturer’s benevolence). However, in regard to this latter aspect, we could argue that a *cautious MAI*, like one that would abstain when in doubt [7], could be considered even more benevolent than a presumptuous one, like most of those currently available.

2 We consider this formulation over the possibly more common one $\frac {m}{2} + 1$ because, as the majority is defined only when m is odd, we require that the formula returns an integers when m is odd.

3 This is expected: the sampling procedure adopted to generate our sample had the goal to avoid significant differences with respect to the original MRNet distribution.

4 However, notice that the vice versa does not hold, as we could still observe high *degree of fineness* with low reliability *ϱ* values, as in the case of raters who consistently assign the right label to the cases but with low confidence (see Eq. 1).

5 It can be noticed that the most basic approach to consider a case *x* compatible with the available data in terms of its closeness to the “center” (or centroid) of the dataset is inadequate. For instance, *x*, while being quite distant from this centroid, could be in a cluster region.

6 This could occur frequently in case of prognostic models: these models could urge doctors choose a specific treatment instead of another, or a more intensive one that those otherwise applied by indicating a poor prospective outcome for a given patient, and hence affects the outcome positively. In so doing, the predictions made on patients admitted after the deployment of the MAI would be wrong, on the pessimistic side.

7 This is but a coarse approximation of their potential performance, as it is plausible that their error rate could vary for many reasons, among which the case complexity.
